# Instruments for evaluating parental support for the practice of physical activity in children and adolescents: A scoping review

**DOI:** 10.1590/1984-0462/2025/43/2024025

**Published:** 2024-11-29

**Authors:** Naildo Santos Silva, Paulo Henrique Guerra, Paulo Felipe Ribeiro Bandeira, Júlio Brugnara Mello, Adroaldo Gaya

**Affiliations:** a Universidade Federal do Rio Grande do Sul, Porto Alegre, RS, Brazil.; b Universidade Federal da Fronteira Sul, Chapecó, SC, Brazil.; c Universidade Regional do Cariri, Crato, CE, Brazil.; d Pontificia Universidad Católica de Valparaíso, Valparíso, Chile.

**Keywords:** Social support, Psychometrics, Review, Apoio social, Psicometria, Revisão

## Abstract

**Objective:**

The objective of this study was to map the instruments used to assess parental support for physical activity and their constructs and psychometric properties.

**Data source:**

A scoping review was conducted, with searches in seven electronic databases and reference lists, covering articles available until April 2022. Original and cross-sectional studies were sought that used questionnaires, inventories or questions to assess parental support for the practice of physical activity and sports by children and adolescents aged 6 to 17 years and that assessed the barriers reported by parents or guardians for not offering support.

**Data synthesis:**

Of the initial 1739 articles, 21 made up the synthesis. From a general perspective, 11 studies from 5 continents used a questionnaire or inventory or question to assess parental support; the majority of the samples evaluated were made up of girls and mothers. The intraclass correlation coefficient was the most used measure to evaluate the reliability of the instruments (10 studies). To assess the reliability of the instruments, Cronbach’s alpha was the most used measure (13 studies).

**Conclusions:**

Only one instrument was constructed respecting the psychometric properties. Authors are advised to consider the importance of following the instrument validity evidence process when developing or adapting instruments.

## RESUMO

**Objetivo:**

Mapear os instrumentos utilizados para avaliar o apoio parental à atividade física, seus construtos e suas propriedades psicométricas.

**Fontes de dados:**

Foi conduzida uma revisão de escopo, com buscas em sete bases de dados eletrônicas e em listas de referências, que abrangeu artigos disponíveis até abril de 2022. Foram buscados estudos originais e transversais que utilizaram questionários, inventários ou perguntas para avaliar o apoio dos pais para a prática de atividade física e esportes de crianças e adolescentes de 6 a 17 anos e que tenham avaliado as barreiras relatadas pelos pais ou responsáveis para o não oferecimento do suporte.

**Síntese de dados:**

Dos 1.739 artigos iniciais, 21 compuseram a síntese. Numa perspectiva geral, 11 estudos, de cinco continentes, utilizaram algum questionário ou inventário ou pergunta para avaliar o suporte parental. A maioria das amostras avaliadas era composta de meninas e mães. O coeficiente de correlação intraclasse foi a medida mais utilizada para avaliar a confiabilidade dos instrumentos (10 estudos). As medidas mais utilizadas para avaliar a consistência interna dos instrumentos foi o Alpha de Cronbach (13 estudos).

**Conclusões:**

Apenas um instrumento foi construído respeitando-se as propriedades psicométricas. Recomenda-se que os autores sigam o processo de evidências de validade de instrumento quando do desenvolvimento ou da adaptação de instrumentos.

## INTRODUCTION

The practice of physical activity in childhood and adolescence is associated with global human development and tends to track to an active life in adulthood.^1,2,3^ However, the current outlook for this stage of life is one of sedentary behavior. Physical activity levels typically decline during adolescence compared to higher activity levels observed in children and teenagers in the distant past.^4^ The level of physical activity usually declines during adolescence.^5,6^ In the distant past, children and teenagers were much more physically active. Currently, low levels of physical activity and high levels of sedentary behavior in childhood and adolescence are striking characteristics worldwide.^7,8,9,10^ Furthermore, these are often established in early childhood.^11,12,13^ Recently, a meta-analysis was carried out that included 43,278 adolescents, and the results indicated that adherence to the WHO guidelines (60 minutes of MVPA every day and muscle and bone strengthening activities 3 times a week) was 19.74% (95% CI between 14.72 and 25.31%).^14^

Thus, the importance of regular long-term physical activity is highlighted, as this practice acts as a preventive factor and reduces excess weight and in the treatment of obesity.^15^ Furthermore, it is associated with improving psychological and emotional well-being, reducing anxiety, depression, and stress.^16^ It is therefore necessary that children and adolescents are supported by a support network that includes, for example, school, along with physical education classes and their parents or guardians. Parental support for physical activity^17,18,19,20^ represents interactions between parents and children in promoting behaviors.^21,22^

Even with such advances, including the recognition of associations between parental support and gender,^23,24,25^ age, barriers, and facilitations perceived by parents in offering support for the practice of physical activity,^26,27,28,29^ it is observed that social support is assessed through different instruments (e.g., questionnaires or recalls), which, in turn, have different constructs and are also, often, more directed to the investigation of specific sports. In this sense, we aim to map the instruments used to assess parental support for physical activity and their constructs and psychometric properties.

## METHOD

This paper is part of a larger project, entitled “Parental support for children and adolescents to practice physical activity,” which aims to develop a questionnaire to assess parental support for physical activity in Brazilian children and adolescents. This study was approved by the Research Ethics Committee of the Federal University of Rio Grande do Sul, under opinion number 6.015.599, with the Certificate of Presentation of Ethical Appreciation number 66638122.0.0000.5347.

Considering the objective of mapping the literature, a scoping review was conducted based on the PRISMA-ScR checklist^30^ and other previous references.^31^ Its protocol was registered on the Open Science Framework platform (Title: Parental support for their children’s physical activity; https://doi.org/10.17605/OSF.IO/B24SU).

This review was designed considering the “population,” “concept,” and “context” framework. Therefore, we established the following inclusion criteria:

1. Original studies that used instruments to assess parental support for the practice of physical activity and sports in children and adolescents aged 6–17 years old;
2. Having assessed the barriers reported by parents or guardians; and
3. Studies published in Portuguese, English, or Spanish, published up to 2021. We considered as instruments the questionnaires, scales, reminders, inventories and interviews used by the studies.

In April 2022, potential studies were searched in seven electronic databases (Lilacs, PubMed, SciELO, Scopus, Sportdiscus, Embase, and Web of Science). The strategies were developed based on the construction developed for Pubmed: (((“Social Support”[Text Word] OR “Support, Social”[Text Word] OR “Social Care”[Text Word] OR “Care, Social”[ Text Word] OR “Parental Support”[Text Word] OR Tangible[Text Word] OR Intangible[Text Word]) AND (“questionnaire”[Text Word] OR “tool”[Text Word] OR “instrument”[Text Word] )) AND (“physical activity”[Text Word] OR “exercise”[Text Word])) AND (valid*[Text Word] OR psychometric*[Text Word] OR reliab*[Text Word]). Controlled health vocabularies via Medical Subject Headings terminology were used for a broad spectrum of results in the different databases. The keywords and combinations of them were organized according to the “population, concept, and context” domains.

This review was developed through five stages:

1. Elaboration of systematic searches and identification of duplicates,
2. Title and abstract assessment,
3. Full-text assessment,
4. Extraction of original data, and
5. Development of the descriptive synthesis.

Stage I involved the joint work of two researchers (NS and PG). Stages II and III were conducted by two independent researchers (NS and JM) previously trained in scoping reviews with the assistance of a third researcher (PG) in order to establish consensus and determine eligibility in cases of disagreement. The main reference for eligibility of the studies was the “population,” “concept,” and “context” framework mentioned above. These two initial stages were conducted on the Rayyan (https://new.rayyan.ai/).

Data extraction (stage IV) was done manually and independently by two researchers (NS and PG) in an electronic spreadsheet organized into three domains:

1. General information (e.g., title; project name; first author’s name and surname; journal in which it was published; year of publication);
2. Contextual information (e.g., location of the study; year of data collection; sample size; sample characteristics; number of individuals according to sex and age group);
3. Methodological information (e.g., sampling technique; instruments; validation measures; statistical analysis).

The descriptive synthesis was drawn up by refining and standardizing the extraction spreadsheet, following its logic.

## RESULTS

**Figure 1:**
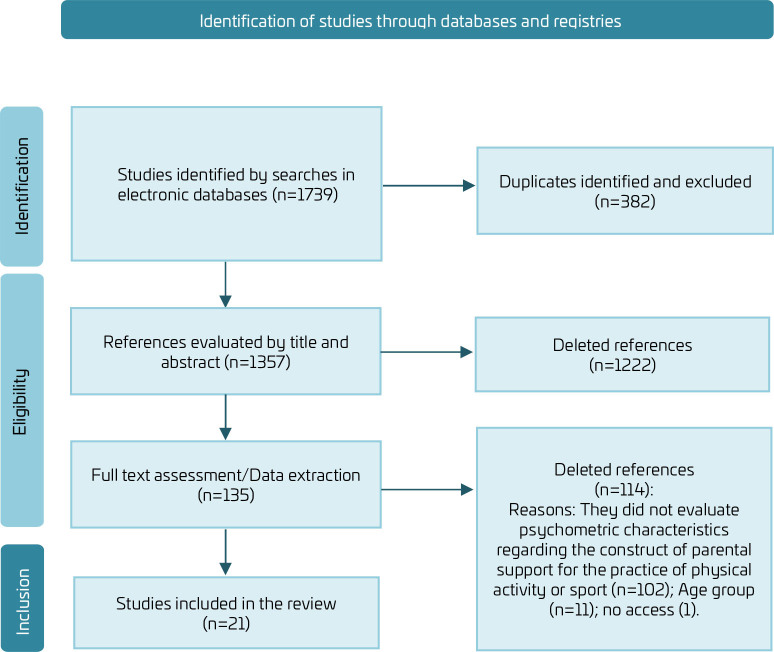
Scope review flowchart.

In all, 1739 potential studies were found (Figure 1). Of these, 1357 studies were assessed by their titles and abstracts. The full-text assessment involved 135 studies. With the exclusion of 114, 18 original studies, reported in 21 papers, adequately met the inclusion criteria and composed the descriptive synthesis of the present review.

Our synthesis is based on studies published in 12 countries on 5 continents (Table 1).^28,29,32-49^ Of the included studies, 15 were conducted with samples of children and/or adolescents (83.3%), and two studies were conducted only with adolescent girls. Different instruments were used for social support assessment (Table 1).

**Table 1:** Descriptive information of included studies.

References	Country	Sample size (mean or age range)	Instrument name
Samples of children and/or adolescents (15 studies)			
Pirasteh et al.^32^	Iran	512 (16)^♀^	Nd
Ries et al.^33^	Luxembourg and Spain	1365 (14; 15)	Self-perception profile for children
Dishman et al.^34^	United States	6778 (12; 14)^♀^	Amherst health and activity study scales
Hendrie et al.^35^	Australia	106 (8)	Nd
Farias Júnior et al.^36^; Farias Júnior et al.^37^	Brazil	248 (14–19); 2755 (16)	The social support for physical activity practices among adolescents’ scale (ASAFA)
Reimers et al.^38^	Germany	196 (12)^†^	Motorik-Modul (MOMO)
Dewar et al.^39^	United States	171 (13)	Nd
Liang et al.^40^	China	273 (10)	Nd
Barbosa Filho et al.^41^	Brazil	1178 (14–19)	Nd
Berki and Piko^42^	Hungary	526 (16)	Source of enjoyment in youth sport questionnaire (SEYSQ)
Loucaides and Tsangaridou^43^	Cyprus	154 (11)	Nd
Fuentesal-García et al.^29^	Spain	455 (11)	Physical activity enjoyment scale (PACES)
Biggs et al.^44^	United States	220 (15)	Support for healthy lifestyle (SHEL)
Mendonça et al.^45^	Brazil	1107 (12)	Social support scale (SSS) and Self-efficacy scale (SES)
Silveira et al.^28^	Brazil	1026 (13)^‡^	Kidscreen-27
Samples of adults (three studies)			
Dominick et al.^46^	United States	37 (≥20)	Instrumental social support for physical activity (ISS)
Wright et al.^47^	United States	283 (37)	Stage-of-change (SOC)
Lin et al.^48^	Taiwan	615 (20–50)	Taiwanese version of activity support scale (ACTS-TW)
Norman et al.^49^	Sweden	242 (≥20)	Parental self-efficacy (PSE)

^♀^female-based sample;

^†^longitudinal analysis sample;

^‡^total sample size. nd: not described.

Table 2 ^28,32,33,36,38-41,45,48^, shows 10 studies that used test-retest instruments to measure reliability. The intraclass correlation coefficient was the most used statistical measure to assess the reliability of the instruments.

**Table 2:** Test-retest reliability data.

References	Results
Samples of children and/or adolescents (nine studies)	
Pirasteh et al.^32^	Mean inter-item correlation (Factor 1: 0.41; Factor 2: 0.40). Pearson test-retest (Factor 1: 0.56; Factor 2: 0.54)
Ries et al.^33^	I think that the more exercise you get, the better (r 0.09); My parents encourage me to play games and sports (r 0.54); My parents give me equipment to play games and sports (r 0.59); My parents really help me to be good at sports (r 0.68); My parents give me financial support for my physical activity participation (r 0.58); My parents take me to the venues of my sporting courses or physical activities (r 0.62)
Farias Júnior et al.^36^	Attitude: ICC 0.89 (95%CI 0.86–0.92); Social support and reasons for engaging in physical activity: ICC 0.75 (95%CI 0.68–0.81); Resources for engaging in physical activity: ICC 0.67 (95%CI 0.58–0.75); Family support: ICC 0.91 (95%CI 0.88–0.93); Friends support: ICC 0.89 (95%CI 0.87–0.92); Access to and appeal of locations for engaging in physical activity: ICC=0.82 (95%CI 0.78–0.87); Safety when engaging in physical activity: ICC 0.67 (95%CI 0.56–0.73); Overall structure and maintenance of neighborhood: ICC 0.75 (95%CI 0.68–0.81)
Reimers et al.^38^	Family support: ICC 0.83. Peer support: ICC 0.67
Dewar et al.^39^	Family support: ICC 0.91 (95%CI 0.88–0.94); Friends support: ICC 0.86 (95%CI 0.81–0.90)
Liang et al.^40^	Family support: ICC 0.86 (95%CI 0.82–0.89); Social support from friends: ICC 0.91 (95%CI 0.88–0.93)
Barbosa Filho et al.^41^	Family support: ICC 0.62 (95%CI 0.56–0.65); Friends support: ICC 0.66 (95%CI 0.62–0.69); School’s teachers support: ICC 0.69 (95%CI 0.65–0.73); Perceived neighborhood environment: ICC 0.62 (95%CI 0.58–0.66); Safety and general state of maintenance: ICC 0.58 (95%CI 0.56–0.62); Access to physical activity facilities: ICC 0.67; (95%CI 0.64–0.71); Physical activity facilities: ICC 0.65 (95%CI 0.61–0.70)
Mendonça et al.^45^	Father’s support (Rho 0.80)*; Mother’s support (Rho 0.76)*; Friends support (Rho 0.75)*; Self-efficacy (Rho 0.72)*
Silveira et al.^28^	Autonomy and parent relation: ICC 0.73 (95%CI 0.66–0.79). Social support and peers: ICC 0.73 (95%CI 0.65–0.78). School environment: ICC 0.71 (95%CI 0.64–0.78)
Samples of adults (one study)	
Lin et al.^48^	Parental modeling (α 0.85); Logistical support (α 0.85); Parental regulation (α 0.75)

*p<0.001. 95%CI: 95% confidence interval; ICC: intraclass correlation; Rho: Spearman’s correlation coefficient.

Table 3 ^28,29,34,35,37,39,41-46,48,49^ presents 13 studies that evaluated the internal consistency of the instruments used. Cronbach’s alpha was the most used statistical measure to evaluate the internal consistency of the instruments. Only one study^28^ used a different analysis, McDonald’s omega coefficient.

**Table 3:** Internal consistency data.

References	Results
Samples of children and/or adolescents (11 studies)	
Dishman et al.^34^	Sixth grade: Family support (α 0.81); Friends support (α 0.75); Eighth grade: Family support (α 0.86); Friends support (α 0.79)
Hendrie et al.^35^	Family support (α 0.79); Familiar activity involvement (α 0.88); Opportunity for role modeling of physical activities (α 0.79)
Farias Júnior et al.^37^	Parents support (α 0.79); Friends support (α 0.90)
Dewar et al.^39^	Friends support (α 0.74); Family support (α 0.78)
Barbosa Filho et al.^41^	Family support (α 0.83); Friends support (α 0.90); School’s teachers support (α 0.84); Perceived neighborhood environment (α 0.75); Safety and general state of maintenance (α 0.73); Access to physical activity facilities (α 0.78); physical activity facilities (α 0.61)
Berki and Piko^42^	Sources of sport enjoyment (α 0.71–0.94)
Loucaides and Tsangaridou^43^	Intangible familiar support (α 0.63); Active parents (α 0.82); Friends support (α 0.75); Tangible parental support (α 0.70); Friends physical activity norms (α 0.66)
Fuentesal-García et al.^29^	Positive enjoyment (α 0.79); Negative enjoyment (α 0.70)
Biggs et al.^44^	Family support (α 0.86); Peer support (α 0.89); Professional support (α 0.95)
Mendonça et al.^45^	Father support (CRI 0.79); Mother support (CRI 0.77); Friends support (CRI 0.78)
Silveira et al.^28^	Social Support and peers (Ω 0.84)
Samples of adults (3 studies)	
Dominick et al.^46^	Instrumental social support (α 0.88); Positive beliefs (α 0.73); Negative beliefs (α 0.74); Normative beliefs (α 0.92); Self-efficacy (α 0.82); Perceived behavioral control (α 0.78)
Lin et al.^48^	Parental modeling (α 0.92); Logistical support (α 0.84); Parental regulation (α 0.78); Total measure (α 0.92)
Norman et al.^49^	Physical activity (α 0.92)

α: alpha; Ω: omega; CRI: combined reliability index.

In Table 4 ^29,35,40,44,48,49^, only six studies used some measure of external validity. Pearson’s correlation was the most used statistical measure. One study^29^ used convergent validity, in which instruments measuring similar constructs are associated as expected.

**Table 4:** Criterion validity data.

Reference	Results
Samples of children and/or adolescents (four studies)	
Hendrie et al.^35^	Family support (r 0.16)
Liang et al.^40^	Family support (r 0.40); Friends support (r 0.35)
Fuentesal-García et al.^29^	Positive enjoyment (Convergent validity 0.82); Negative enjoyment (Convergent validity 0.75)
Biggs et al.44	Family support (bivariate correlation 0.08); Peer physical activity support (bivariate correlation 0.21)*; Professional physical activity support (bivariate correlation 0.23)*
Samples of adults (two studies)	
Norman et al.^49^	Moderate-to-vigorous physical activity average mins/day weekend (r 0.13); moderate-to-vigorous physical activity average min/day, weekday outside of 8 am to 4 pm (r 0.19)^†^
Lin et al.^48^	A one-unit increase in parental support increased the odds of sufficient physical activity approximately 2.44 times

*p<0.05;

^†^p<0.01. r: Pearson correlation.

## DISCUSSION

Based on data from 21 original cross-sectional studies, this review summarized the instruments used to assess parental support for physical activity and identified their constructs and psychometric properties. The synthesis suggested that few studies utilized instruments with comprehensive external validity measures and that the majority of studies focused on samples of adolescent girls.

Recognizing the link between physical activity and parental support and assessing physical activity levels among Brazilians have posed significant challenges due to the continued high costs associated with objective measurement methods. Consequently, indirect measures have become the predominant approach, with parental support typically evaluated through questionnaires, inventories, or interviews.

To be deemed reliable and meet rigorous standards, an instrument must adhere to established standards and undergo thorough evaluation for both internal and external validity. In this sense, the instrument must undergo evaluations for content, criterion, and construct validity phases. Currently, the Standards for Educational and Psychological Testing^50^ have been the most current recommendations used to validate and adapt instruments.

The global interest in parental support for children’s physical activity and sports is evident, as reflected in studies from various countries and continents identified in this review. However, with the exception of the study,^48^ none of the other studies^29,38,42,48-50^ used questionnaires that presented all the external validity measures (content validity, criterion validity, and construct validity) recommended by the American Psychological Association. These present only one of these measures. This review highlighted in its synthesis the predominance of studies that do not meet all validity evidence processes.

Many of these studies focused solely on querying parental support for physical activity without addressing crucial steps such as translating the instrument from the source to the target language, synthesizing translated versions, conducting expert analysis, evaluating the target population, or adhering to the recommended content, criterion, and construct validity processes outlined in the Standards for Educational and Psychological Testing.^50^ Among the studies reviewed, only one^48^ undertook these steps and, based on the culture of their sample, added new items to their questionnaire.

Over the years, the construct of parental support for physical activity and/or sports has been investigated globally through various methodologies. Initially, a scale was developed to measure family and friend support for exercise and eating behaviors in children and adolescents aged 8–16 years.^51^ Five items were proposed to assess support from friends (e.g., “exercised with me,” encouraged me to exercise), and 15 items were proposed to assess support from family. The first five items are the same as the friend’s support. Items such as “discussed exercise” and “helped plan activities around my exercise” are items that differentiate the assessment of family support from support from friends.

Other results^52^ assess parents and children in the United States. Through surveys administered to parents and self-reports from children, various factors were investigated, including race, parental education level, engagement in physical activities and sports, leisure time habits, parental physical activity, peer influence, and neighborhood characteristics like park access and distance from home. Therefore, questions such as how often the family encourages, provides transportation to the child, watches the child practice physical activity, or talks about the importance of practicing physical activity were applied.

Based on these aforementioned studies, other authors identified in this review applied the same five questions and/or adapted the items, however, without considering the validity processes recommended by the Standards for Educational and Psychological Testing.^50^

Although parental support for practicing physical activity and/or sports since childhood is recommended, most of the studies included in this review only involve samples of adolescents.^28,29,32,34,36,37,39,41,44,53^ When encouraged to be physically active from childhood, children have a great chance of becoming healthy adults.^53,54^ When children are encouraged to be physically active from an early age, they have a greater chance of becoming healthy adults. In this sense, parental support during childhood becomes relevant, as studies show that, as children mature, parental support for physical activity decreases and may even be replaced by peer support. Consequently, the earlier physical activity is encouraged and becomes a habit, the greater the health benefits for children and adolescents will be, for example, weight control, a lower chance of developing some types of cancer and chronic diseases such as diabetes, high blood pressure, and heart disease.^55^

Generally, most boys are more physically active compared to girls. Nevertheless, in the present review, the majority of studies involved samples (mostly) of girls^32,34,36,43^ and adolescents.^28,32-34,36,39,41,42,44,46^

Most studies involved samples of adolescents and investigated other behaviors along with support, such as the intake of soft drinks, vegetables, and sweets. Given this limitation, the relevance of parental support may end up being overshadowed. Furthermore, during adolescence, peer support becomes more efficient for adolescents. In this sense, it is recommended to analyze studies with children since in early childhood, parents are considered role models for their children and this habit can become a lifelong trend.

Another limitation observed was that the minority of studies were able to assess both parents (father and mother), which is essential for understanding which source provides the most support. Overall, most studies evaluated psychometric aspects, with only one^48^ assessing all properties, including test–retest reliability, internal consistency reliability, and criterion validity. Cronbach’s alpha was the most used statistical measure to evaluate the internal consistency of the instruments. Despite being widely used, Cronbach’s alpha presents problems as it considers the factor loadings of the instruments to be equal, that is, having the same relevance (tau-equivalence).

In conclusion, it was identified through this work that, although there are international and national studies that have used instruments to assess parental support for the practice of physical activity and/or sports in children and adolescents, only one instrument was constructed respecting psychometric properties. It is recommended that authors when developing or adapting instruments follow the instrument validity evidence process proposed by the Standards for Educational and Psychological Testing.^50^ Currently, these are the recommendations.

In this sense, the following evidence will be considered:

a) content validity,
b) criterion validity, and
c) construct validity.
